# Amyloid-bodies in the evolution of malignancies

**DOI:** 10.1371/journal.pone.0353464

**Published:** 2026-07-09

**Authors:** Alexander Michael Grunfeld, Michael Bokros, Karelia Paz, Daniel Bilbao, Stephen Lee

**Affiliations:** 1 Sheila and David Fuente Graduate Program in Cancer Biology, Miller School of Medicine, University of Miami, Miami, Florida, United States of America; 2 Department of Biochemistry and Molecular Biology, University of Miami Miller School of Medicine, Miami, Florida, United States of America; 3 Sylvester Comprehensive Cancer Center, University of Miami Miller School of Medicine, Miami, Florida, United States of America; 4 Medical Scientist Training Program, University of Miami Miller School of Medicine, Miami, Florida, United States of America; 5 Department of Pathology and Laboratory Medicine, University of Miami Miller School of Medicine, Miami, Florida, United States of America; Shanghai Jiao Tong University, CHINA

## Abstract

Tumorigenesis depends on the capacity for cancer cells to survive in the presence of various environmental stressors. An emerging paradigm in the study of cancer cell stress responses is their regulation by membrane-less intracellular compartments known as biomolecular condensates. While there has been considerable progress in our understanding of biomolecular condensates in cancer *in vitro*, a paucity of evidence remains for their presence and function in settings which more closely reflect *in vivo* tumor physiology. In this study, we use human tissues and an *in vivo* orthotopic mouse model to study the role of the Amyloid-body, a stress-induced condensate, in tumorigenesis. We present methodology to visualize Amyloid-bodies in tumors by multiple immunohistochemical approaches in addition to a semi-automated analysis pipeline. Analysis of multiple tumor types reveals that Amyloid-bodies are detectable at all tumor grades and stages, to varying degrees, and negatively correlate with the cell proliferation marker Ki-67. Finally, in an orthotopic mouse model of breast cancer, we show that silencing long noncoding ribosomal intergenic spacer RNA (rIGSRNA) involved in Amyloid-body formation accelerates tumorigenesis *in vivo*. Together, these results suggest that Amyloid-body formation occurs in human cancers, further establishing the physiological relevance of biomolecular condensates.

## Introduction

Cancer development is a multistep process which involves the gradual accumulation of genetic and epigenetic alterations resulting in aberrant cellular signaling [1]. Within the last decade, there has been a rapid increase in studies investigating how the mechanisms of this dysregulation are related to biomolecular condensates [2]. Biomolecular condensates are functional, membraneless compartments composed of concentrated subsets of proteins and nucleic acids. Condensates vary in characteristics such as their localization, composition, and material properties, all of which contribute to differences in function [3]. Most condensates identified to date form by liquid-liquid phase separation (LLPS), a rapid and reversible process whereby subsets of macromolecules concentrate into droplet-shaped foci via weak, multivalent interactions [4,5]. Condensates formed by LLPS exhibit ‘liquid-like’ behavior: they undergo cycles of fusion/fission and their constituent molecules dynamically exchange with their surroundings [5–7]. Liquid-like condensates are believed to concentrate molecules to facilitate biochemical reactions in response to cellular cues [7–15]. In contrast to liquid condensates, there exists a smaller subset of condensates whose constituents are immobile and thus described as ‘solid-like’ [16–18]. The solid-like condensates identified to date share a common theme of operating as storage hubs of RNA, proteins, and in some cases, organelles, to drive cellular dormancy in various settings [16,19]. Both liquid and solid condensates have been observed in various species across phyla and in cultured cells responding to different stimuli highlighting their ubiquitous nature [20].

We, and others, have reported that environmental stressors induce physiological amyloidogenic programs that convert elements of the liquid-like nucleoli into the solid-like condensate Amyloid-body; reversible nuclear membrane-less compartments enriched in immobilized proteins. Amyloid-bodies display several properties typically associated with amyloid deposits observed in pathological settings, most prominently Alzheimer’s disease. This includes staining with multiple amyloidophilic dyes, assembly into fibrillar structures, resistance to detergents and proteinase K digestion, and yellow-green birefringence under polarized light in vivo [21]. A short peptide domain that forms fibrils in vitro with the classical amyloid X-ray diffraction pattern facilitates the capture for immobilization of several proteins by Amyloid-bodies [19]. These amyloidogenic assemblies are seeded by a class of inducible noncoding RNAs transcribed form stimuli-specific loci of the ribosomal intergenic spacer (rIGSRNA). The three principal rIGSRNA consists of the heat shock-induced rIGS_16_RNA/rIGS_22_RNA and extracellular acidosis rIGS_28_RNA named by where they are transcribed within the ribosomal intergenic spacer [22]. During stress, the rIGSRNA recruits Amyloid-body proteins by electrostatic interactions into transient intranucleolar foci with liquid-like properties [23]. These foci undergo rapid amyloidogenic liquid-to-solid maturation to form Amyloid-bodies, a process facilitated by TENT4b-synthesized long poly(A) tailed rRNA that operates as polyanionic stimulators of amyloidogenesis [24]. On stimuli termination, Amyloid-bodies are disassembled by the heat shock chaperone pathway releasing proteins that return to their original steady state localization. Thus, Amyloid-bodies share properties with the amyloid-fold that distinguish these solid-like condensates from the many liquid-like membraneless compartments.

While Amyloid-bodies share properties with pathological amyloids, they do differ in their functional relevance. One of the major functions associated with Amyloid-bodies is the suppression of cellular metabolism via protein immobilization. Proteomic and photobleaching analyses revealed that Amyloid-bodies capture for immobilization an array of proteins, including catalytic subunits of the DNA synthesis machinery and other key regulators of cellular metabolism [19,22]. Taken together with studies showing that exposing cells *in vitro* to Amyloid-body inducing stressors coincides with a state of proliferative arrest and decreased metabolic activity (e.g., restricted rRNA synthesis, ATP utilization), these data raised the hypothesis that Amyloid-body biogenesis is linked to these changes in cellular activity [25–27]. Disrupting Amyloid-body formation *in vitro* by silencing rIGSRNA enabled continued cell proliferation and metabolism even during stress [19]. Together, these data suggest that protein immobilization in Amyloid-bodies facilitates entrance into a dormant-like cellular state in response to extracellular stressors.

While there has been considerable progress in our understanding of condensates *in vitro*, their roles in physiological and pathological settings, including cancer, are not fully understood [28]. Since Amyloid-bodies are induced by extracellular acidosis, a hallmark feature of the tumor microenvironment [29], human tumors are an ideal system to study these condensates in a clinically relevant context. Using the unique biochemical properties of the amyloid fold, we present methodology to detect Amyloid-bodies in tissues by multiple immunohistochemical staining approaches. Using these techniques, we show that Amyloid-bodies are readily detectable in human tumors from multiple tissue types and explore how Amyloid-bodies correlate with measures of tumor aggressiveness, cellular activity, and disease prognosis.

## Results

### Amyloid-bodies are a common feature of solid human tumors

**Fig 1:**
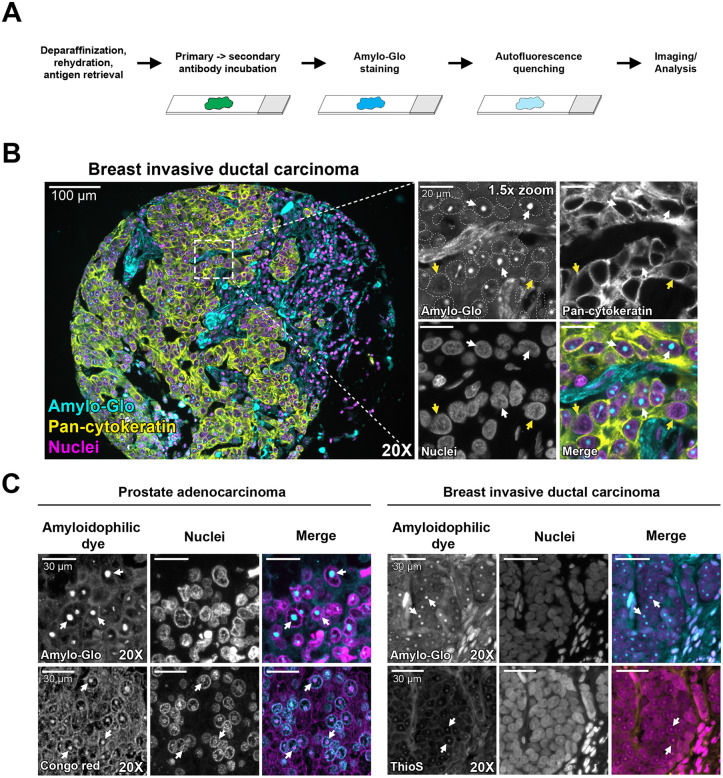
Human tissues contain Amyloid-bodies. A). Summary of procedure used to stain FFPE tissue sections with the amyloidophilic dye Amylo-Glo. B). Representative image of a breast invasive ductal carcinoma tumor stained for Amyloid-bodies (Amylo-Glo, cyan), pan-cytokeratin (yellow), and nuclei (magenta). White and yellow arrows indicate cells which do or do not contain Amyloid-bodies, respectively. C). Serial sections of prostate adenocarcinoma or breast invasive ductal carcinoma tumors were stained with Amylo-Glo, Congo red, or Thioflavin S.

Amyloid-bodies display unique properties including staining with various amyloidophilic dyes, formation of electron-dense fibers, and the immobilization of central participant proteins of cellular metabolism. These features distinguish Amyloid-bodies from the array of liquid-like condensates that typically do not display these amyloidogenic properties. Extracellular acidosis stimulates the formation of Amyloid-bodies in essentially all tested primary and established cancer cell lines studied so far [19,22,30]. As extracellular acidosis is a common feature of solid tumors, we hypothesized that malignant lesions would provide an ideal pathology to study the existence and function of these condensates in human cancer. To that end, we developed a staining protocol (described in materials and methods) using the amyloidophilic dye Amylo-Glo [31], which identified subnuclear Amylo-Glo positive foci in formalin-fixed paraffin-embedded (FFPE) sections of human tumors from various tissues of origin (Fig 1A, 1B, S1A). Tumor sections consistently displayed a mixed population of foci-positive and foci-negative cells, highlighting the specificity of Amylo-Glo staining (Fig 1B). Importantly, these foci also stained positively with the amyloidophilic dyes Congo red or Thioflavin S, suggesting they contain broadly detectable amyloidogenic features (Fig 1C) recognized by multiple dyes. To assess potential contributions of residual tissue autofluorescence, we compared serial sections of a prostate adenocarcinoma tumor which were either left unstained or stained with Amylo-Glo alone (Fig S1B). Here, the unstained section showed minimal background signal when imaged with the same exposure times as the Amylo-Glo stained section. Imaging the unstained section at a higher exposure revealed apparent residual autofluorescence, however, we were unable to detect background signal in these images resembling the Amylo-Glo positive foci. We note that the higher contrast of these foci observed in sections stained with Amylo-Glo compared with the other tested dyes is likely related to the use of a tissue autofluorescence quenching reagent (Vector TrueView Autofluorescence quenching kit), which, in our hands, is compatible with Amylo-Glo but not Congo red or Thioflavin S.

We further evaluated the amyloid-like properties of these foci. A feature of the amyloid-fold shared by Amyloid-bodies in cultured cells is resistance to proteinase K digestion. We treated serial sections of a breast invasive ductal carcinoma (IDC) tumor with either PBS or Proteinase K followed by staining for nuclei (NUCLEAR-ID), Amyloid-bodies (Amylo-Glo), and nucleoli (B23) (Fig S2A). Here, Amyloid-bodies were still detectable in the proteinase K treated section despite the loss of B23 signal, further demonstrating the amyloid-like properties of these solid-like condensates. Next, we tested if these foci show apple-green birefringence under polarized light when stained with Congo red [32]. While investigators demonstrated that nucleolar foci with Amyloid-body-like properties exhibit apple-green birefringence in brain tissue [21], we only observed low signal intensity in tumors (Fig S2B). Finally, we tested whether amyloidophilic dyes exhibit competitive binding at these foci. Congo red and thioflavin T (ThT) have been suggested to bind to amyloid fibrils via similar binding modes [33]. We therefore stained serial sections with either Congo red alone or sequentially with Congo red followed by a molar excess of ThT. Sequential staining resulted in several ThT positive foci with relatively weak Congo red staining compared to the section stained with Congo red alone (Fig S2C). Together, these data are suggestive of additional amyloidogenic features exhibited by these Amylo-Glo positive foci.

**Fig 2:**
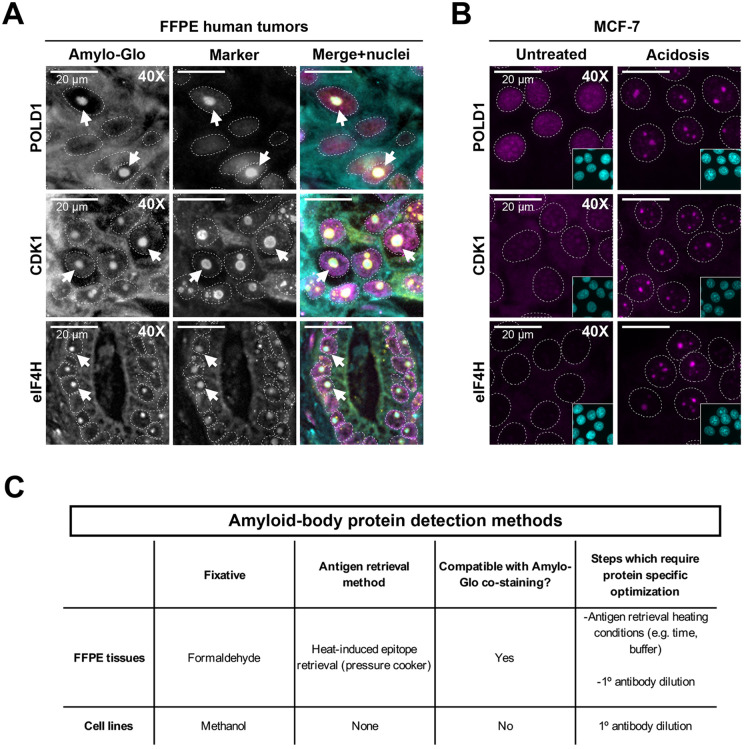
Amyloid-bodies in tissues are enriched in Amyloid-body target proteins. A). Immunofluorescent staining of breast invasive ductal carcinoma tumors for Amyloid-bodies (Amylo-Glo), Amyloid-body target proteins POLD1, CDK1, or eIF4H, and nuclei. B). Immunofluorescent staining of the same Amyloid-body proteins in (A) in MCF-7 cells exposed to extracellular acidosis. C). Comparison of methods to detect Amyloid-body proteins in FFPE tissue sections versus cultured cell lines.

To further establish these Amylo-Glo positive foci as *bona fide* Amyloid-bodies, we next tested whether they colocalize with known Amyloid-body target proteins [19]. Immunohistochemical staining of a breast cancer tissue microarray (TMA) revealed that Amylo-Glo positive foci were enriched with the catalytic subunit of DNA polymerase delta (POLD1), cyclin dependent kinase 1 (CDK1), and eukaryotic translation initiation factor 4H (eIF4H), (Fig 2A). These proteins are similarly captured in Amyloid-bodies *in vitro* in MCF-7 breast cancer cells exposed to extracellular acidosis (Fig 2B), further highlighting the similarity between Amyloid-bodies observed in tissues and their *in vitro* counterparts. One technical requirement we note regarding the detection of Amyloid-body proteins *in vitro* is the use methanol as a fixative (Fig 2C). The enrichment of most Amyloid-body target proteins within these condensates is not visible in formaldehyde-fixed cells (Fig S3A). To overcome this limitation in FFPE tissues, which are fixed in formalin, we found the method of antigen retrieval noticeably impacts Amyloid-body protein signal. Specifically, the use of high-pressure heating in a pressure cooker, but not boiling, successfully unmasks the epitopes of these proteins in Amyloid-bodies (Fig S3B). Taken together, the Amylo-Glo positive foci we observe in tissues resemble Amyloid-bodies both in their amyloidogenic properties and protein composition and will henceforth be referred to as such.

**Fig 3:**
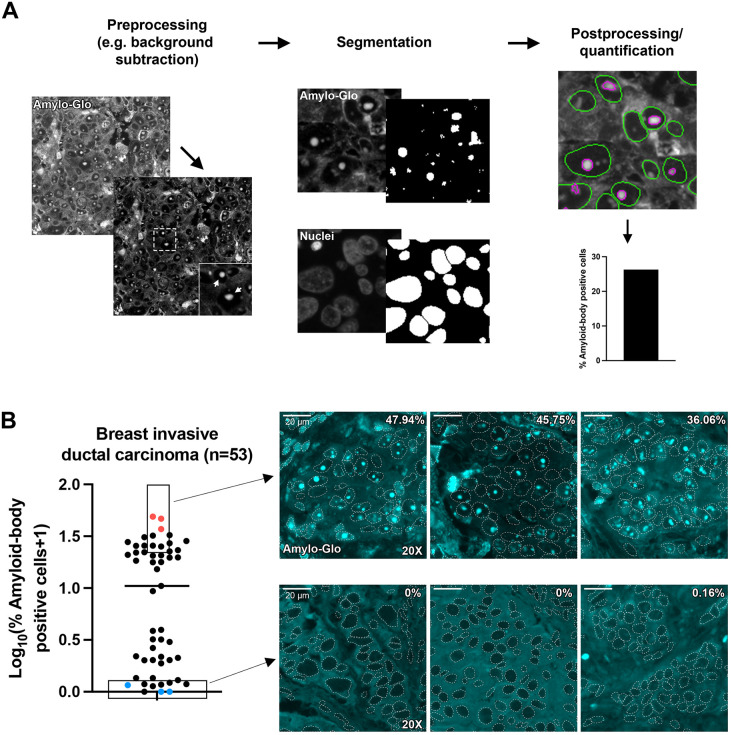
Semi-automated analysis of Amyloid-bodies in tissues. A). Overview of image analysis pipeline. Following preprocessing steps such as background subtraction, Amylo-Glo and nuclei channels were thresholded using Ilastik and Cellpose, respectively. Thresholded images were then imported into CellProfiler to perform final quantification of percent Amyloid-body positive cells. B). Representative example of analysis of a sample of breast invasive ductal carcinoma tumors (data plotted on a log (y + 1) scale) with images (right panels) of the Amylo-Glo staining for the corresponding top and bottom three tissues. Nuclei are represented by dotted lines.

**Fig 4:**
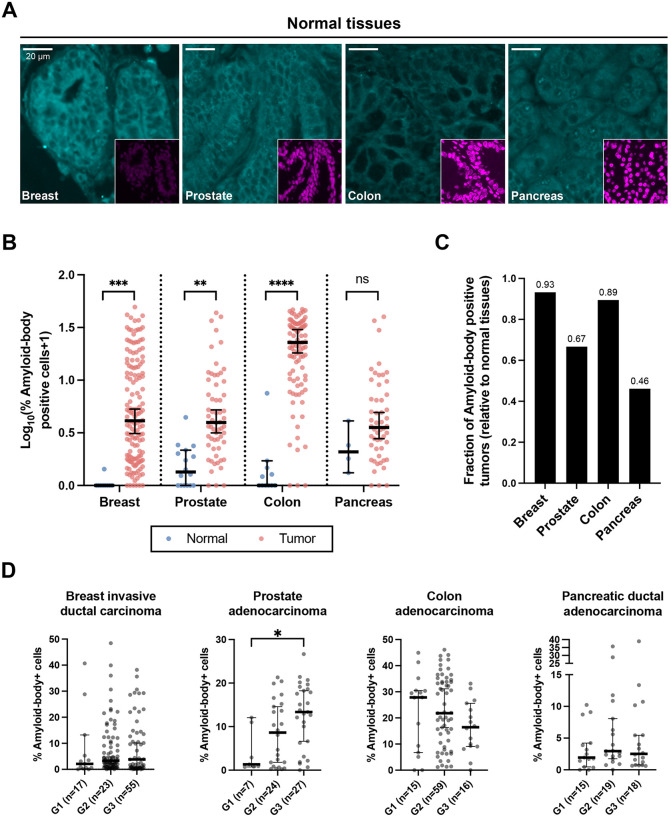
Analysis of Amyloid-bodies across tumor grade. A). Representative images of normal tissues from each tissue type tested B). Comparison of percent Amyloid-body positive cells between primary tumors and normal tissues for the indicated tissue types (data plotted on a log (y + 1) scale). Tumor types tested were breast invasive ductal carcinoma, prostate adenocarcinoma, colon adenocarcinoma, and pancreatic ductal adenocarcinoma. Tissues were analyzed by a Kruskal-Wallis test followed by a Dunn’s multiple comparison post hoc test. The frequencies of Amyloid-body positive cells were found to be statistically significant between tissue groups (H(7)=144.9, p < 0.0001). Post hoc pairwise comparisons within each tissue type showed significant differences between healthy controls and breast invasive ductal carcinoma (p = 0.0007), prostate adenocarcinoma (p = 0.0083), and colon adenocarcinoma (p < 0.0001) tumors. ****, p < 0.0001; ***, p = 0.0001, **, p = 0.0012. Data are presented as median ± 95% confidence interval C). The fraction of Amyloid-body positive tissues, defined as containing a frequency of Amyloid-body positive cells > 95^th^ percentile relative to the normal control of that tissue type. D). Comparison of Amyloid-bodies from tumors in (B) categorized by tumor grade. Grade groups were compared by a Kruskal-Wallis test followed by a Dunn’s multiple comparison post hoc test. In prostate adenocarcinoma tumors (H(2)=7.704, p = 0.0212), a significant difference in percent Amyloid-body positive cells was found between grade 1 and grade 3 tumors (p = 0.0347).

In addition to staining methodology, we developed semi-automated image analysis pipelines to quantify the percentage of Amyloid-body positive cells in a given tumor section (Fig 3A–3B, S4A-S4B). Using this pipeline, we first analyzed TMAs of primary tumors and their normal tissue counterparts. Each tumor type showed a heterogeneous distribution of Amyloid-body frequency, containing tissues which we qualitatively categorized as Amyloid-body negative, strongly Amyloid-body positive, or belonging to an intermediate phenotype characterized either by a small number of cells containing prominent Amyloid-bodies or a larger number of cells containing smaller, low-intensity foci (Fig S5). In contrast, normal tissues contained very few and in most cases, no Amyloid-bodies (Fig 4A–4B). Among the tumor groups for each tissue type, we found that breast IDC tumors contained the highest proportion of Amyloid-body positive tissues, defined for each tissue as having a frequency of Amyloid-body positive cells greater than the 95^th^ percentile of the corresponding normal tissue (Fig 4C). We next analyzed Amyloid-bodies in each tumor type with respect to histologic grade. Here, we found significant differences between tumor grade for prostate cancer, specifically between grade 1 and grade 3 prostate tumors (Kruskal-Wallis test H(2)=7.704, p = 0.0347), but not in any of the other tested tumor types (Fig 4D). Together, these data suggest that the presence of Amyloid-bodies is predominantly specific to diseased tissues but independent of histologic grade.

### Amyloid-bodies exist within variable histologic patterns

**Fig 5:**
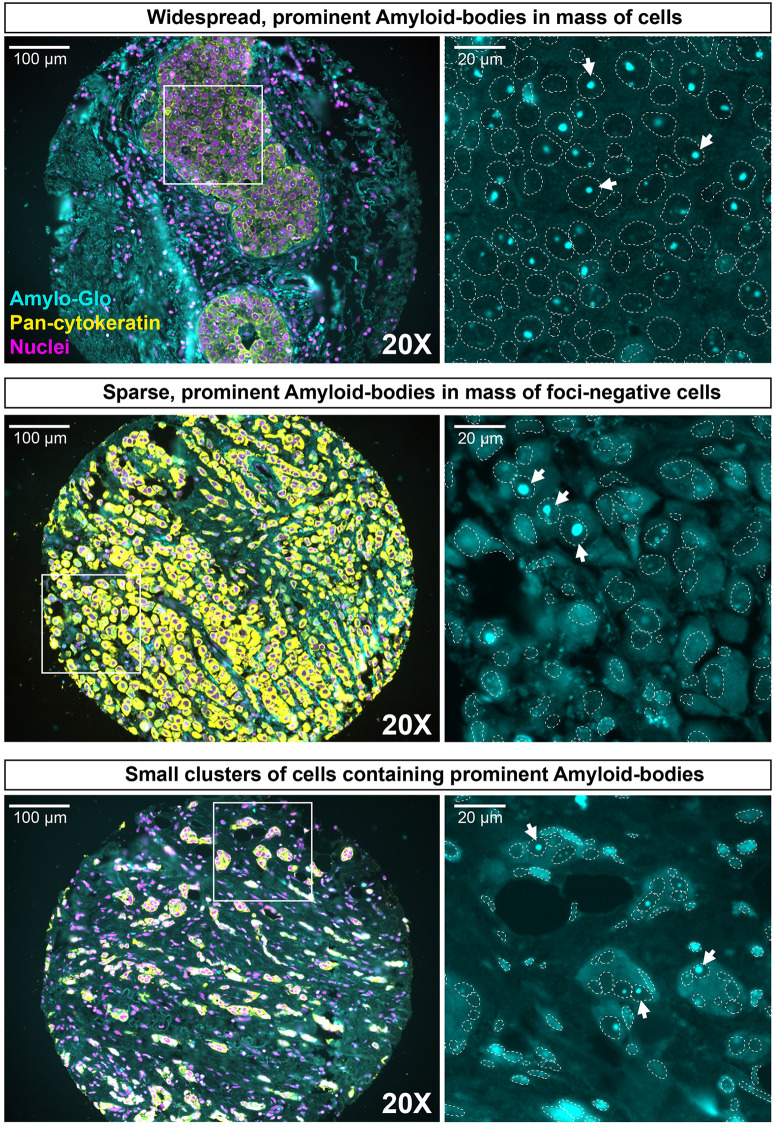
Amyloid-bodies can exist in various histologic patterns. Representative examples of different patterns of Amyloid-body positive cells within various tissue histology patterns. Top panel: Tumor cells arranged as a cohesive mass, in which a majority of cells are Amyloid-body positive. Middle panel: Tumor cells arranged in sparse clusters, where tumor cells in each cluster are Amyloid-body positive. Bottom panel: Tumor cells arranged in a mass, similar to the top panel, however, only a few cells are Amyloid-body positive and are surrounded by a much larger number of Amyloid-body negative cells.

We noticed that Amyloid-body positive cells could be present as part of various tissue-wide histologic patterns (Fig 5). Amyloid-body positive cells could be evenly distributed throughout a large tumor nodule, in sparse clusters, or as individual cells within a larger mass of Amyloid-body negative cells. This suggests that Amyloid-body formation is not limited to tumors of a particular size. In addition, that Amyloid-bodies can be found in a small number of cells surrounded by a mass of Amyloid-body negative cells may be suggestive of underlying genetic or environmental heterogeneity.

### Amyloid-bodies in tumor cells negatively correlate with cell proliferation

**Fig 6:**
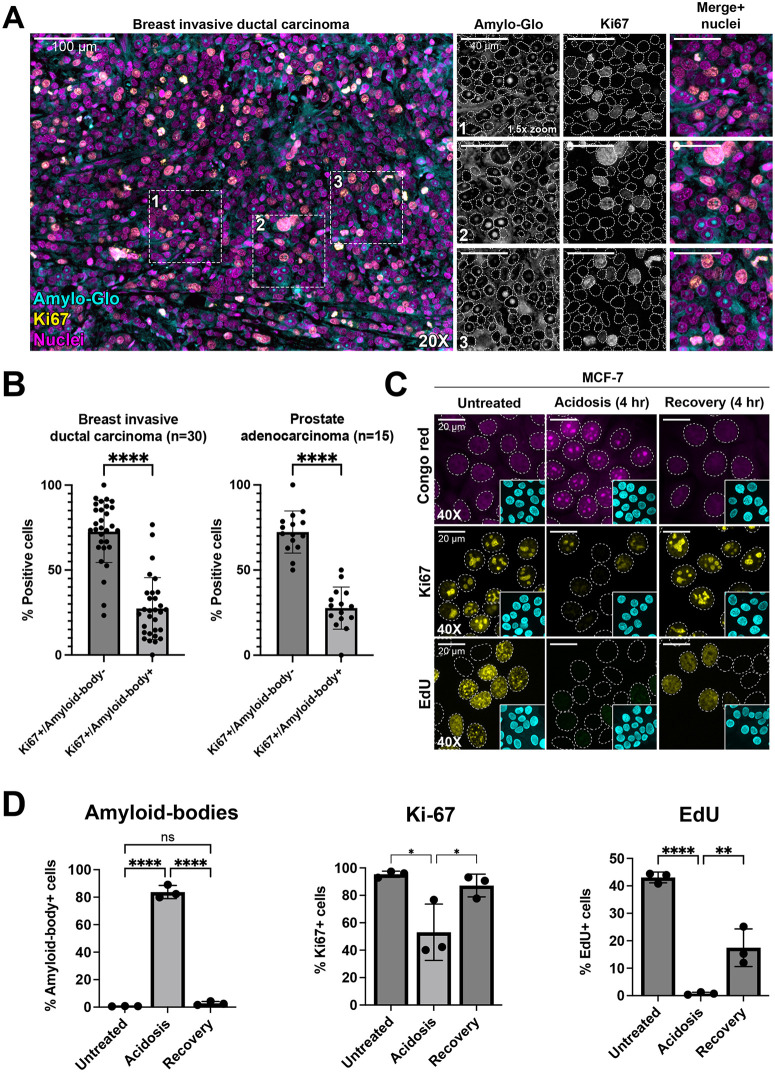
Amyloid-bodies negatively correlate with cell proliferation. A). Breast invasive ductal carcinoma tumor stained for Amyloid-bodies (Amylo-Glo), Ki-67, and nuclei. B). Breast invasive ductal carcinoma and prostate adenocarcinoma TMAs were stained for Amyloid-bodies and Ki-67. Among cases containing both Amyloid-body positive cells and Ki-67 expression, Ki-67 positive cells were classified as Amyloid-body positive or negative. Ki-67 + /Amyloid-body+ and Ki-67 + /Amyloid-body- cell populations were compared by a two-tailed student’s t-test. ****, p < 0.0001. C). MCF-7 cells were maintained in basal growth conditions (21% O_2_, pH 7.4), treated under hypoxia-acidosis for 4 hr (1% O_2_, pH 6.0), or treated under hypoxia-acidosis and then placed back into basal growth conditions (recovery) for 4 hr. Following indicated treatments, cells were fixed and stained for the indicated markers. D). Quantification of percent-positive cells for Amyloid-bodies, Ki-67 expression, and EdU incorporation for images shown in C). For each marker, treatment conditions were compared by a one-way ANOVA followed by a Tukey’s post-hoc test. ****, p < 0.0001. **, p = 0.0051. *, p = 0.0136 (Ki-67 untreated vs. acidosis), p = 0.0346 (Ki-67 acidosis vs. recovery). Data are presented as the mean ± s.d. of n = 3 biological replicates.

*In vitro*, Amyloid-body formation coincides with halted cell proliferation in cells cultured under hypoxia-acidosis. Thus far, the Amyloid-bodies we observe in tissues share several characteristics with their *in vitro* counterparts with respect to protein composition and amyloidogenic properties. Therefore, we next asked whether these Amyloid-body positive cells also exhibit a phenotype consistent with reduced cellular activity. To this end, we co-stained TMAs for Amyloid-bodies and the cell proliferation marker Ki-67. Within Amyloid-body positive breast and prostate tumors, the majority of Ki-67 positive cells were Amyloid-body negative (two-tailed student’s t-test, p < 0.0001; Fig 6A, 6B). We recapitulated these findings *in vitro* in MCF-7 breast cancer cells induced to form Amyloid-bodies by exposure to hypoxia-acidosis (Fig 6C). Here, Ki-67 expression significantly decreased under hypoxia-acidosis (one-way ANOVA; F(2,6)=9.156, p = 0.015; untreated vs acidosis p < 0.0161; Fig 6D), consistent with previous work showing decreased cell proliferation under these conditions [19]. Furthermore, similar to our results in tissues, we observed a remaining population of cells which were notably positive for both Amyloid-bodies and Ki-67. To further investigate the phenotype of these Amyloid-body positive/Ki-67 positive cells, we additionally assessed the proliferative capacity of these cells via labeling with a brief (30 min) pulse of 5-ethynyl-2’-deoxyuridine (EdU) to measure active DNA synthesis [34]. Here, we observed a decrease in EdU incorporation in cells treated under hypoxia-acidosis (one-way ANOVA, F(2,6)=80.03, p < 0.0001; untreated vs acidosis p < 0.0001), suggesting that DNA synthesis is inhibited under acidosis (Fig 6D). Together, these results suggest that Amyloid-bodies correlate with a non-proliferative state.

### Loss of amyloid-bodies in overt metastases

**Fig 7:**
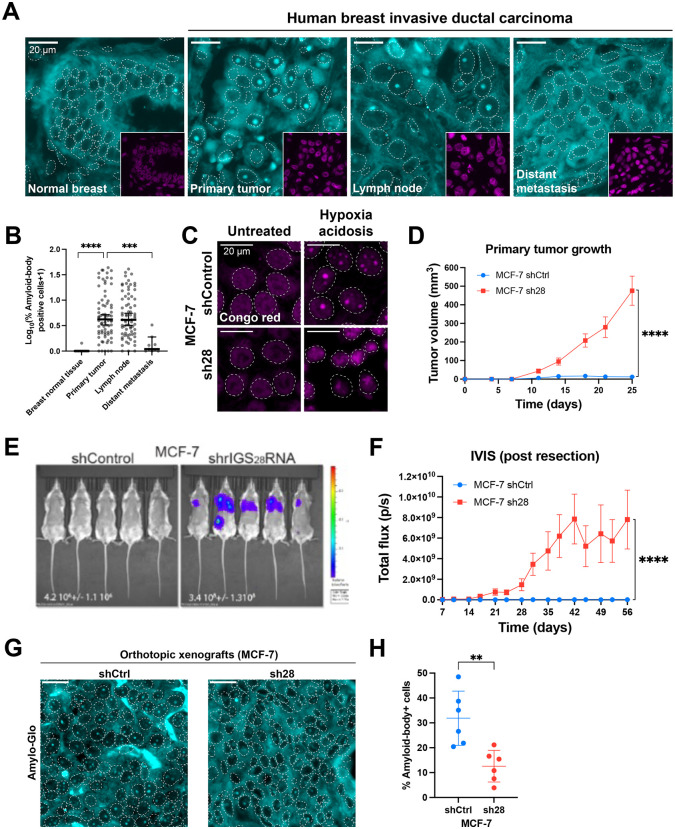
Amyloid-bodies are lost in metastatic disease. A). Comparison of Amyloid-bodies in normal breast tissue, breast IDC primary tumors, breast IDC lymph node metastases, and breast IDC distant metastases. B). Quantification of tissues in (A). Tissue samples were compared by a Kruskal-Wallis test (H(3)=36.46, p < 0.0001) followed by a Dunn’s post-hoc test. Quantification of normal breast tissue is the same as that used in Fig 4A. ****, p < 0.0001. ***, p = 0.0004. Normal breast tissue n = 10, Primary tumors n = 74, Lymph node metastases n = 74, distant metastases n = 9. C). Congo red staining of MCF-7 shCtrl or shIGS_28_RNA cells following growth under basal growth conditions (untreated, 21% O_2_, pH 6.0) or hypoxia-acidosis (1% O_2_, pH 6.0). D). Caliper measurements of MCF-7 shCtrl and sh28 primary tumor growth following orthotopic implantation into NSG mice. ****, p < 0.0001. E) IVIS imaging of mice following primary tumor resection. F) Quantification of IVIS imaging. ****, p < 0.0001. G) Amylo-Glo staining of MCF-7 shCtrl and sh28 primary tumors. Nuclei are represented by dotted outlines. H). Quantification of Amyloid-bodies in xenografts. **, p = 0.0038.

Secondary lesions (i.e., metastasis) are a principal cause of mortality in cancer [35]. In a cohort (n = 9) of breast invasive ductal carcinoma distant metastases, Amyloid-bodies were sparse in these tissues relative to primary breast tumors and lymph node metastases (Fig 7A, 7B). We note, however, interpretation of these data are limited by small sample sizes, particularly of the distant metastases, and the mixing of paired tissues (paired primary tumors and lymph node metastases) with unpaired tissues. Nevertheless, these data raised the question if metastatic progression may be related to a decreased or lost capacity to form Amyloid-bodies. To test this, we performed orthotopic xenograft assays using MCF-7 breast adenocarcinoma cells which stably express a control short hairpin RNA (shRNA) or an shRNA targeting rIGS_28_RNA (referred to as MCF-7 shControl and sh28 cells, respectively). Relative to shControl, MCF-7 sh28 cells show impaired Amyloid-body biogenesis induced by hypoxia-acidosis *in vitro* (Fig 7C) and accelerated primary tumor growth *in vivo* in a mouse flank xenograft model [19]. To assess the *in vivo* effects of silencing rIGS_28_RNA in a more clinically relevant context, MCF-7 shControl and sh28 cells were orthotopically implanted in the mammary fat pad of NOD-SCID-gamma (NSG) mice without exogenous estradiol supplementation. In contrast to MCF-7 shControl cells, silencing rIGS_28_RNA enabled rapid growth of large primary tumors (two-way ANOVA, p < 0.0001, Fig 7D) and the formation of distant metastases (two-way ANOVA, p < 0.0001, Fig 7E, 7F). Amylo-Glo staining confirmed the presence of Amyloid-bodies in shControl primary tumors (Fig 7G). Amyloid-bodies were also detectable in sh28 primary tumors, albeit at a significantly lower frequency relative to shControl (two-tailed student’s t-test p = 0.0038, Fig 7H). Together, these results suggest that rIGS_28_RNA regulate mechanisms that suppress tumor growth and metastasis.

## Discussion

In this study we highlight the Amyloid-body, a solid-like biomolecular condensate with amyloidogenic properties, as a clinically relevant feature of human cancers. We describe methodology to detect Amyloid-bodies in FFPE tissue sections and, using semi-automated image analysis, show that Amyloid-bodies are detectable in multiple solid tumor types arising from distinct tissues of origin. These Amyloid-bodies exhibit similarities to their *in vitro* counterparts including positive staining with amyloidophilic dyes, resistance to proteinase K digestion, sequestration of Amyloid-body target proteins, and the predominant absence of Ki-67 expression.

Studies on condensate biology are most frequently performed *in vitro* using purified proteins and cell lines. However, these systems do not necessarily recapitulate how a condensate behaves in a human tissue within its native microenvironment. The nucleolus is perhaps the most widely studied condensate in tissues, where increases in nucleolar number and prominence are established hallmarks of tumor histology [36–38]. Outside of the nucleolus, however, there are few studies which have presented similar evidence for other condensates in human tissues [39–44]. Stress granules are one example of a stress-induced condensate which has been shown to exist in tissues, however, these findings have been met by a recent study suggesting otherwise [45]. Our work presents multiple methods of identifying Amyloid-bodies in tissues, either by staining with amyloidophilic dyes or immunostaining for Amyloid-body target proteins. We find these methods are affected by technical considerations such as choice of amyloidophilic dye, the specific protocol used to perform the dye staining, and in the case of detecting Amyloid-body target proteins, the antigen retrieval method. In this study, we predominantly detected Amyloid-bodies by staining with Amylo-Glo, a styrylbenzene derivative which was demonstrated in amyloid plaques to exhibit comparable specificity to Congo red and Thioflavin S while also producing higher intensity staining [31]. We found the Amylo-Glo staining protocol is amenable to modifications such as treatment with autofluorescence quenching reagents which improved the dye’s staining specificity. Nevertheless, the specificity of amyloidophilic dyes remains debatable [32]. In addition, another group showed that in vivo Amyloid-body-like assemblies display the classical apple-green birefringence under polarized light following Congo red staining while we only detected low signal, which may be due to limitations of our microscope system [21]. We therefore cannot exclude the possibility that dyes such as Amylo-Glo may also be detecting other types of nucleolar amyloidogenic foci or nucleolar stress bodies which are distinct from Amyloid-bodies.

Amyloid-bodies appeared across multiple tumor types, each of which were characterized by a variable distribution of Amyloid-body positive cells. These data were obtained from TMAs where all tissues were included on a single slide, making it unlikely this heterogeneity was observed due to technical differences in tissue staining. One possible explanation for this variability is differences in the tumor microenvironment, such that localized gradients of extracellular acidosis (or other stressors) act as regional stimuli to induce Amyloid-bodies. On a molecular level, differences between tumors may arise from differential activity of RNA polymerase II that prevents induction of rIGSRNA [46], activation of heat shock chaperone that stimulates Amyloid-body disassembly [19], or loss of TENT4B that prevents the formation of long poly(A) tails on rRNA necessary for phase transition [24]. In addition, the absence of Amyloid-bodies in distant metastases could be due to differences in treatment history and microenvironment. It should be noted that our study only includes n = 9 distant metastasis due to difficulties of obtaining clinical samples and as such these results remain preliminary. Elucidating whether these differences correlate with Amyloid-body frequency, or if other pathways are involved, remains an open question for future studies.

In addition to positive amyloidophilic dye staining, we showed that Amyloid-bodies in tumor cells colocalize with known Amyloid-body target proteins. These proteins include POLD1, CDK1, and eIF4H, which regulate DNA synthesis, cell cycle progression, and translation, respectively [47–49]. Furthermore, expression of the proliferation marker Ki-67 was predominantly associated with Amyloid-body negative cells in breast and prostate tumors. *In vitro*, induction of Amyloid-body formation via extracellular acidosis similarly correlated with a decrease in Ki-67 expression and EdU incorporation. Subsequent recovery in basal growth media coincided with the loss of Amyloid-bodies and recovery of these markers towards untreated levels. Taken together, we speculate that Amyloid-bodies in tumors correlate with a non-proliferative state, possibly by sequestering one or multiple proteins involved in regulating cell proliferation. However, whether this non-proliferative state is reversible as shown *in vitro* remains to be seen. Future studies will aim to assess other similarities between Amyloid-bodies in tissues and their *in vitro* counterparts such as whether the microenvironments of these tissues are consistent with acidosis. Interestingly, recent studies have reported the appearance of Congo red-positive nucleoli in response to other stimuli such as chemotherapy drugs [50,51]. This raises the possibility that Amyloid-bodies in tissues may alternatively or additionally be associated with other cell fates such as senescence or apoptosis.

*In vivo* experiments in this study showed that silencing rIGS_28_RNA promotes a highly malignant phenotype in an orthotopic xenograft mouse model of breast cancer. MCF-7 sh28 cells showed accelerated primary tumor growth and a lower frequency of Amyloid-body positive cells relative to shControl, suggesting that silencing rIGS_28_RNA inhibits Amyloid-body formation *in vivo*. While correlative, we speculate the increased primary tumor growth in sh28-derived xenografts may be related, in part, to deficient Amyloid-body formation via silencing rIGS_28_RNA. In addition, sh28 cells notably exhibited the capacity to form overt metastases. Metastasis is a multistep cascade consisting of invasion of tissue surrounding the primary tumor, intravasation and survival in the circulation, and colonization and proliferation in a distant target organ, among other steps [52]. Mechanistic links between rIGS_28_RNA and/or Amyloid-bodies and these processes are unclear and will be an important direction to explore in future studies. In addition, a limitation of the rIGS28RNA datasets is the difficulty of using CRISPR to remove expressing loci owing to the large number of rDNA cassettes in the genome (>450 repeats) and the chromatin-associated nature of these lncRNAs that prevents rescue experiments. Aside from its role in regulating Amyloid-body formation, other biological functions of rIGS_28_RNA largely remain unknown. One recent study identified pyrimidine-rich noncoding transcript (PNCTR), an IGS-derived lncRNA containing sequence elements of rIGS_28_RNA [53]. PNCTR is highly expressed in cancer cells, including MCF-7s, relative to non-transformed cells. However, unlike rIGS_28_RNA, PNCTR is substantially longer (>10 kb versus ~300 bp for rIGS_28_RNA), expressed in unstressed cells, and instead appears to play a pro-tumorigenic role. Nevertheless, this highlights the possibility for rIGS_28_RNA containing RNA species to perform biological functions beyond regulating Amyloid-body formation.

Overall, our findings provide evidence for Amyloid-bodies as a clinically relevant marker of tumor cells in human tissues. This study contributes to a growing body of work which examines condensates in human tissues across varying stages of disease progression. As there is an ongoing need for these types of studies in the field of condensate biology, we anticipate additional examples highlighting their clinical significance in varying disease contexts will become evident.

## Materials and methods

### Cell lines and treatments

MCF-7 cells were purchased from ATCC (cat #HTB-22, RRID:CVCL_0031) and grown in DMEM supplemented with 10% FBS and 1% penicillin-streptomycin. MCF-7 cells stably expressing rIGS_28_RNA shRNA (sh28) or a scrambled control shRNA (shCtrl) were previously described [19,22]. At least one day prior to the start of all treatments, 500,000 cells were seeded in 35 mm plates on 22x22 mm #1 coverslips. Acidosis experiments were performed by gently rinsing cells three times with DMEM without bicarbonate (Thermo Fisher Scientific #12100046) adjusted to pH 6.0 and then incubating cells for the indicated times in an H35 Hypoxystation at 37 ºC set to maintain 1% O2 and 5% CO2 in a nitrogen-balanced atmosphere. Recovery was performed following treatments by aspirating acidic media, replacing with pH 7.4 growth media, and then incubating cells at 37 ºC, 21% O_2_ for the indicated times. Cell lines were authenticated by STR profiling and regularly tested for mycoplasma contamination (Applied Biological Materials #G238).

### Tissue sections

Tissue microarrays of breast invasive ductal carcinoma, prostate adenocarcinoma, colon adenocarcinoma, and pancreatic ductal adenocarcinoma were purchased from TissueArray LLC (BR1507, BR1508, BRE1121, PR753a, PR1921c, CO1201, PA961f) and the Cooperative Human Tissue Network (CHTN) and Cancer Diagnosis Program, which are funded by the National Cancer Institute. Tissue samples of breast invasive ductal carcinoma primary tumors and breast invasive ductal carcinoma distant metastases were collected retrospectively from the University of Miami under approval by the University of Miami Institutional Review Board (protocol #20110663). For all tissues used in this study (i.e., tissues obtained from the University of Miami, TissueArray LLC, CHTN), any available patient data associated with these tissues were accessed for research purposes between June 1^st^, 2021 and September 10^th^, 2025. Patient information was fully anonymized prior to receipt of tissues. Information that could identify individual patients was not accessible either during or after data collection. For tissues obtained from the University of Miami, a waiver of informed consent was approved in accordance with 45 CFR §46.116(f)(3). Other investigators may have received specimens from the same subjects.

### Antibodies

For immunocytochemistry and immunohistochemistry, primary antibodies and their used dilutions were: mouse α-B23 (MilliporeSigma #B0556, 1:50), mouse α-Cytokeratin (Dako #M351529-2, RRID:AB_2132885, 1:100), rabbit α-eIF4H (Cell Signaling Technology #3469, RRID:AB_2096038, 1:100), rabbit α-Ki67 (Abcam #ab16667, RRID:AB_302459, 1:100), rabbit α-POLD1 (Abcam #ab186407, RRID:AB_2921290, 1:100), rabbit α-phospho-4E-BP1 (Cell Signaling Technology #2855S, RRID:AB_560835, 1:100)

Secondary antibodies and their used dilutions were: Goat α-Rabbit Alexa Fluor™ 488 (Thermo Fisher #A11008, RRID:AB_143165, 1:250), Goat α-Mouse Alexa Fluor™ Plus 488 (Thermo Fisher #A32723, RRID:AB_2866489, 1:250).

### Immunocytochemistry

Coverslips were rinsed once with 1X phosphate-buffered saline (1X PBS, pH 7.4) and fixed for 15 minutes at room temperature with 4% formaldehyde in 1X PBS. After fixation, coverslips were washed 3x5 min with 1X PBS, permeabilized 1x5 min in 1X PBS + 0.5% Triton X-100, blocked for 1 hr with 1X PBS + 10% horse serum, and incubated for 2 hrs at room temperature or overnight at 4 ºC with primary antibody diluted in 10% normal goat serum (10% NGS) (Thermo Fisher Scientific #50062Z). Following primary antibody incubation, coverslips were washed 3x5 min with PBS + 0.1% Tween-20 (1X PBST) and incubated for 1 hr at 37 ºC with the appropriate secondary antibody diluted in 10% NGS. Coverslips were then washed 3x10 min with 1X PBST, incubated for 10 min with 10 μg/mL Hoechst 33342 in 1X PBS, and mounted with Fluoromount™ (MilliporeSigma #F4680).

For immunofluorescent detection of Amyloid-body proteins, cells were fixed with −20 ºC methanol for 10 min instead of 4% formaldehyde. All other steps of the immunostaining protocol were performed as described above.

### Immunohistochemistry

Formalin-fixed paraffin-embedded sections were baked at 60 ºC for 30 minutes, deparaffinized in xylenes (2x5 min, 1x10 min), rehydrated in a series of graded ethanols (3x3 min 100% ethanol, 2x3 min 90% ethanol, 1x2 min 70% ethanol, 1x2 min 50% ethanol, 1x2 min 30% ethanol), and then rehydrated for 10 min in deionized water (diH_2_O). Antigen retrieval was performed in a pressure cooker (Instant Pot Duo 7-in-1 Electric Pressure Cooker) for 10 min at high pressure (10.2–11.6 psi) in either sodium citrate (10 mM sodium citrate, pH 6.0) or Tris-EDTA (10 mM Tris, 1 mM EDTA, pH 9.0) buffer. Following antigen retrieval, the pressure cooker was left undisturbed for 15 min to allow pressure to naturally release and then manually vented to release remaining pressure. Slides were cooled at room temperature for 1 hr, washed 3x5 min with 1X PBS, permeabilized 2x10 min with 1X PBS + 0.3% Triton X-100, blocked for 1 hr in 10% NGS, and incubated overnight at 4 ºC with primary antibody diluted in 10% NGS. Following primary antibody incubation, slides were washed 3x5 min in 1X PBST and incubated for 1 hr at 37 ºC with the appropriate secondary antibody diluted in 10% NGS. Slides were washed 3x10 min with 1X PBST, counterstained with 1:500 NUCLEAR-ID® Red DNA stain (Enzo Life Sciences ENZ-52406) in 1X PBST for 30 min at room temperature, and then washed 1x5 min with 1X PBS. If staining with Amylo-Glo, following nuclear counterstaining, slides were washed 1x2 min with ddH_2_O, incubated with 1:100 Amylo-Glo (Biosensis #TR-300-AG) in 0.9% NaCl for 15 min at room temperature, and washed 1x5 min with 0.9% NaCl. After all staining steps, slides were treated with Vector TrueVIEW® Reagent (VectorLabs #SP-8400–15) for 5 min at room temperature to quench tissue autofluorescence. Slides were then washed 1x5 min with 1X PBS and mounted in VECTASHIELD Vibrance (VectorLabs H-1800).

### Amyloidophilic dye staining

All cells to be stained with amyloidophilic dyes (Congo red, Amylo-Glo) were fixed in 4% formaldehyde in 1X PBS for 15 min, washed three times with 1X PBS, and permeabilized for 5 min with 1X PBS + 0.5% Triton X-100.

To stain cells with Congo red, following permeabilization and washing, cells were counterstained with 10 μg/mL Hoechst 33342 (Thermo Fisher #H3570) in 1X PBS for 10 min. Cells were then washed 1x5 min in 1X PBS, incubated with 0.05% Congo red in ddH_2_O for 10 min, washed 3x5 min with ddH_2_O, and mounted in 5% glycerol (diluted in ddH_2_O).

To stain cells with Amylo-Glo, following permeabilization and washing, cells were counterstained with 1:500 NUCLEAR-ID® Red DNA stain in 1X PBS for 30 min at room temperature. Cells were washed 1x5 min with 1X PBS, washed 2x2 min with ddH_2_O, and incubated with 1:100 Amylo-Glo (Biosensis #TR-300-AG) in 0.9% NaCl for 10 min. Cells were washed 1x5 min with 0.9% NaCl, rinsed briefly in ddH_2_O, and mounted in 5% glycerol (diluted in ddH_2_O).

To perform sequential staining of Congo red followed by Thioflavin T (ThT), following dewaxing and rehydration, sections were stained with 0.3 mM Congo red in ddH_2_O for 15 min, washed 1x5 min in ddH_2_O, and then incubated for 15 min with 30 mM ThT in ddH_2_O. Sections were washed 2x5 min in ddH_2_O and mounted in 5% glycerol.

### Proteinase K digestion

Serial sections of FFPE tissues were dewaxed, rehydrated, and subjected to antigen retrieval in 10 mM sodium citrate buffer as described above. After cooling sections to RT, sections were washed 3x5 min in 1X PBS, permeabilized 2x10 min in 1X PBS + 0.3% Triton-X100, and then incubated for 20 min at 37 ºC in either 1X PBS alone or 1X PBS + 20 µg/mL Proteinase K (New England Biolabs P8107S). Sections were washed 2x5 min in 1X PBS and then immunostained as described above.

### EdU labeling

EdU labeling was performed using the Click-iT™ EdU Cell Proliferation Kit for Imaging (Thermo Fisher #C10337). Following indicated treatments, cells were incubated for 30 min at 37 ºC with 40 μM EdU diluted in growth medium, rinsed once with 1X PBS, and fixed with 4% formaldehyde for 15 min. EdU detection was then performed according to the manufacturer’s instructions.

### Microscopy and image analysis

All images were acquired with a Keyence BZ-X810 All-in-One Fluorescence Microscope equipped with Plan Apochromat 4X, 20X, and 40X objectives. The following filter cubes were used: DAPI (excitation: 360/40 nm, emission: 460/50 nm), GFP (excitation: 470/40 nm, emission: 525/50 nm), TexasRed (excitation: 560/40 nm, emission: 630/75 nm). Brightness/contrast adjustments were uniformly applied across all images.

Tissue sections were analyzed as follows. First, images were preprocessed in Fiji [54] using the subtract background (rolling ball radius = 100) and remove outliers (radius = 1.5, threshold = 50) functions. Nuclei were segmented using Cellpose [55] and Amyloid-bodies were segmented from the Amylo-Glo channel using a pixel classification model trained in ilastik [56]. Specifically, this model was trained on three representative cases each of Amyloid-body negative, intermediate, and high tissues. Remaining tissues were then batch analyzed using this model. Thresholded images of the nuclei and Amylo-Glo channels were then imported into CellProfiler to analyze percent positive cells [57]. Briefly, thresholded images of the Amylo-Glo and nuclear channels were first converted to objects. The pan-cytokeratin image was additionally thresholded and the ‘maskobjects’ module was used to remove any nuclei outside of the boundaries of pan-cytokeratin staining. Then, Amylo-Glo objects outside of the nuclei were removed (also using the ‘maskobjects’ module) and remaining Amylo-Glo objects were additionally filtered based on size and eccentricity parameters to eliminate remaining cytoplasmic artifacts. The ‘relateobjects’ module was then used to relate the Amylo-Glo objects to the nuclear objects and finally obtain the percentage of Amyloid-body positive cells. Following this analysis, tissues belonging to the top or bottom 10% of percent-positive cells were then binned as strongly positive or negative, respectively.

Images of *in vitro* experiments with MCF-7 cells were analyzed using a similar analysis pipeline as above. However, segmentation of Amyloid-bodies from Congo red images was instead performed in cellprofiler using the ‘EnhanceOrSuppressFeatures’ module followed by thresholding using Otsu’s method [58].

### Animal studies

Animal studies were performed with the help of the Cancer Modeling Shared Resource (CMSR, RRID: SCR_022891) at the University of Miami. All animal procedures were approved by the University of Miami Institutional Animal Care and Use Committee (IACUC protocol #20-113). As required by the animal protocol, all staff involved in animal experiments were trained on all aspects of the approved protocol, including specific procedures, species handling and observation. NOD Scid Gamma (NSG) female mice were obtained from the Jackson Laboratory (Stock No. 002374) and bred inhouse for one generation. MCF-7 cells were transduced with lentiviruses expressing GFP-luciferase (pCDH-MSCV-IRES-Luciferase-EF1-GFP), and successful transduction confirmed by imaging cells on cell imager (Zoe, Biorad) with a GFP filter. After transduction of cells, GFP-positive cells were sorted and purified by FACS, expanded in vitro, and used in the in vivo experiments.

For xenograft model generation, 1  ×  10^6^ control or sh28 labeled MCF-7 cells were resuspended in Corning^®^ Matrigel^®^ Basement Membrane Matrix and PBS in a 1:1 ratio and injected orthotopically (mammary fat pad) in female adult NSG mice (*n*  =  10 per group; 20 animals in total). Animal health and behavior were monitored at least once daily. Primary tumor growth was monitored biweekly using caliper and the in vivo imaging system (IVIS Spectrum, Revvity) until day 25 from injection, when they were surgically resected. Tumor volumes were calculated as follows: Length x Width x Width/2. Primary tumor resection was performed under approved anesthesia procedures. After the surgical procedure, animals were monitored and left undisturbed in a warm, quiet, clean place until they recovered. Post-operative care included pain management, antibiotic treatment, and animal monitoring for signs of infection, illness or distress. All animals recovered from the surgery and metastatic tumor burden was subsequently evaluated by IVIS until day 102. For IVIS imaging, 10 min prior to imaging, mice were injected intraperitoneally with d-luciferin (Perkin Elmer #760504) at a dose of 150 mg/kg and image acquisition performed under anesthesia. Animals reaching endpoint, defined as more than 20% weight loss or changes in health status including any signs of pain, distress, were immediately euthanized (sh28 group, n = 8). Two animals from the sh28 group died before reaching endpoint and the rest of the animals (control group; n = 10) were euthanized at day 102. Following approved animal protocols, euthanasia was performed by CO2 inhalation followed by cervical dislocation.

### Statistical analysis

All statistical analyses were performed in GraphPad Prism (RRID:SCR_002798). *In vitro* experiments were performed on three biological replicates. Error bars represent standard deviation. Data were first assessed for normality by a Shapiro-Wilk test before performing statistical analysis. Experiments comparing two groups were analyzed by performing an unpaired two-tailed student’s t-test with the significance level set to p < 0.05. Experiments comparing three or more groups were analyzed by performing a one-way ANOVA with a post hoc Tukey’s HSD or Kruskal-Wallis test followed by Dunn’s post hoc test corrected for multiple comparisons. Descriptions of statistical tests used and p-values are provided in the figures.

## Supporting information

S1 Fig Amyloid-bodies are detectable in multiple solid tumor types. A). Representative images of FFPE sections of colon adenocarcinoma, prostate adenocarcinoma, lung adenocarcinoma, and pancreatic ductal adenocarcinoma tumors which were found to contain Amylo-Glo positive foci. B). Serial sections of a prostate adenocarcinoma tumor were stained with Amylo-Glo or left unstained. The Amylo-Glo stained section (top row) contains bright Amylo-Glo positive foci (white arrows) in cells of the malignant glands (outlined in dotted yellow lines) compared to the unstained section (middle row) imaged at the same exposure. Imaging the unstained section at a high exposure (bottom row) shows the presence of cytoplasmic background fluorescence with minimal background staining of the foci seen in the Amylo-Glo stained section. Exposure times used for each channel are indicated in the top right corner.
(PDF)

S2 Fig Amyloid-like properties of Amyloid-bodies. A). FFPE serial sections of a breast invasive ductal carcinoma tumor were incubated with 1X PBS or 20 µg/mL proteinase K for 20 min at 37 ºC and stained for Amyloid-bodies (Amylo-Glo) and nucleoli (B23). B). Polarized light microscopy of a breast invasive ductal carcinoma tumor stained with Congo red. C). FFPE serial sections of a prostate adenocarcinoma tumor stained with 0.3 mM Congo red alone or sequentially stained with 0.3 mM Congo red followed by 30 mM Thioflavin T (ThT).
(PDF)

S3 Fig Amyloid-body target protein detection is affected by fixation and antigen retrieval methods. A). Comparison of fixation methods on Amyloid-body protein detection in cultured cell lines. MCF-7 cells were grown in basal growth conditions (21% O_2_, pH 7.4) or in hypoxia-acidosis (1% O_2_, pH 6.0) for 3 hours, fixed, and stained for POLD1. B). Comparison between boiling and pressure cooking as antigen retrieval methods to detect the Amyloid-body target protein POLD1 (yellow) in FFPE tissues.
(PDF)

S4 Fig Amylo-Glo segmentation performed by machine-learning based pixel classification. A). Detailed schematic showing how segmentation of Amylo-Glo images was performed in Ilastik. Using the pixel classification toolkit, three classes of pixels were manually defined as the Amyloid-body (yellow line), nucleoplasmic background (blue line), or cytoplasmic background (pink line). After training ~6 images with this classification scheme, the resulting pixel classification model was applied to all remaining images in the data set to produce a combined thresholded image of all three pixel classes. This image was then imported into fiji to isolate the “Amyloid-body” thresholded image to import into CellProfiler for additional processing and quantification. B). Comparison of Amylo-Glo segmentation performed in ilastik versus traditional thresholding algorithms (Otsu’s and moments). Nuclei (cyan outlines) and the thresholding output (yellow) are shown as overlays on the original Amylo-Glo image. The Amylo-Glo example used is a representative image with Amyloid-body negative cells. Compared to the thresholded images produced by Otsu’s method and moments, the ilastik segmentation is more specific and less likely to threshold cytoplasmic signal which may later be detected in the nucleus as false-positive signal.
(PDF)

S5 Fig Amyloid-body phenotypes across tumor type. Representative images of tumors which are Amyloid-body negative, strongly positive, or intermediate in phenotype (containing few cells with prominent Amyloid-bodies or a larger number of cells with small and/or low intensity Amyloid-bodies).
(PDF)
