# Supplementary figures and images for: Amyloid-bodies in the evolution of malignancies
Source: PLoS One. 2026 Jul 9;21(7):e0353464. doi: 10.1371/journal.pone.0353464 (PMC13349100; doi:10.1371/journal.pone.0353464)

**A**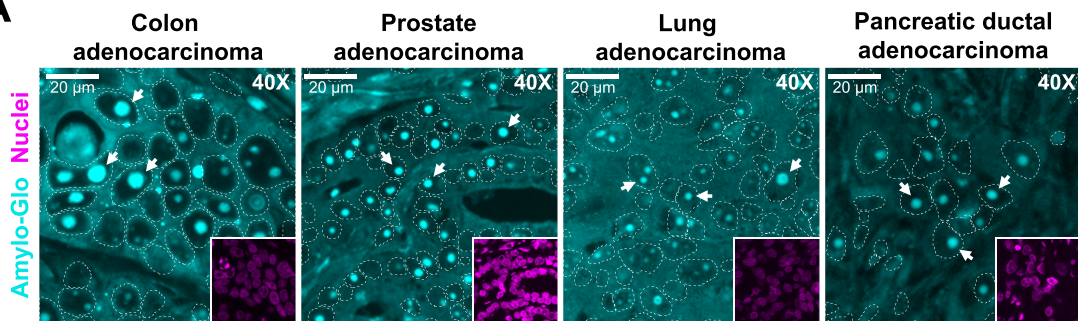**B**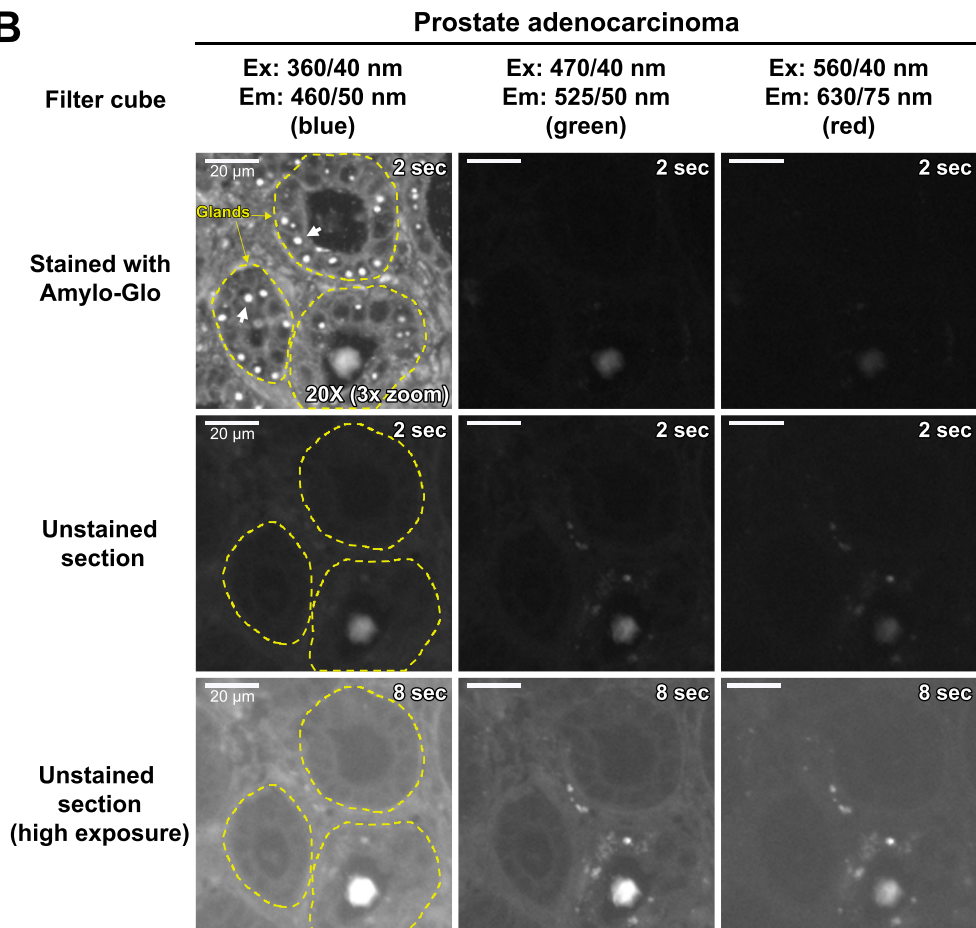

Supplement: S1 Fig — A). Representative images of FFPE sections of colon adenocarcinoma, prostate adenocarcinoma, lung adenocarcinoma, and pancreatic ductal adenocarcinoma tumors which were found to contain Amylo-Glo positive foci. B). Serial sections of a prostate adenocarcinoma tumor were stained with Amylo-Glo or left unstained. The Amylo-Glo stained section (top row) contains bright Amylo-Glo positive foci (white arrows) in cells of the malignant glands (outlined in dotted yellow lines) compared to the unstained section (middle row) imaged at the same exposure. Imaging the unstained section at a high exposure (bottom row) shows the presence of cytoplasmic background fluorescence with minimal background staining of the foci seen in the Amylo-Glo stained section. Exposure times used for each channel are indicated in the top right corner. (PDF) [file pone.0353464.s001.pdf]

**A**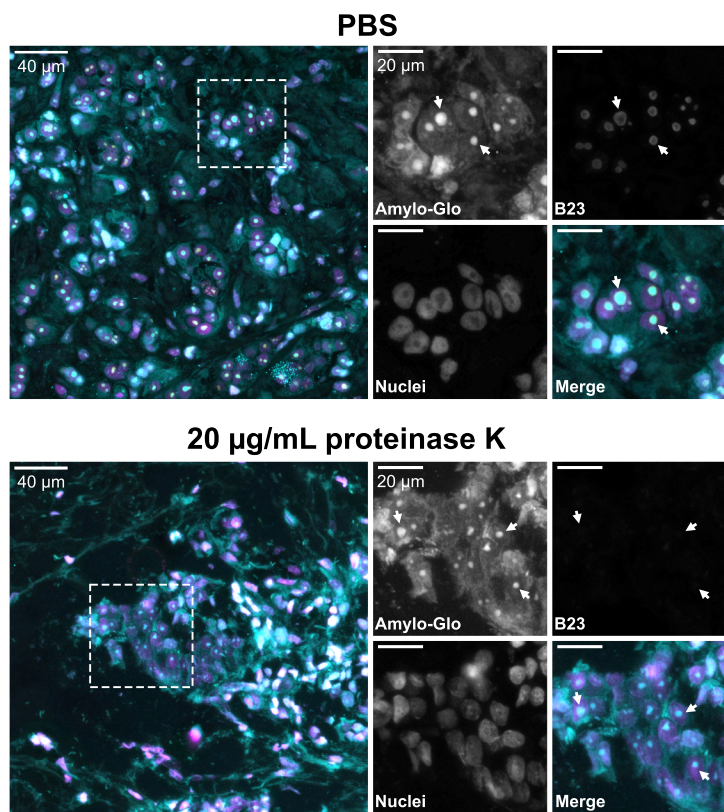**B**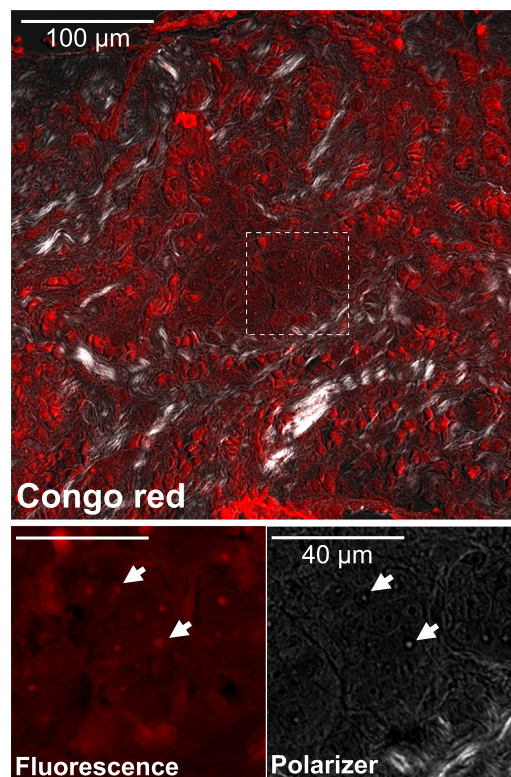**C**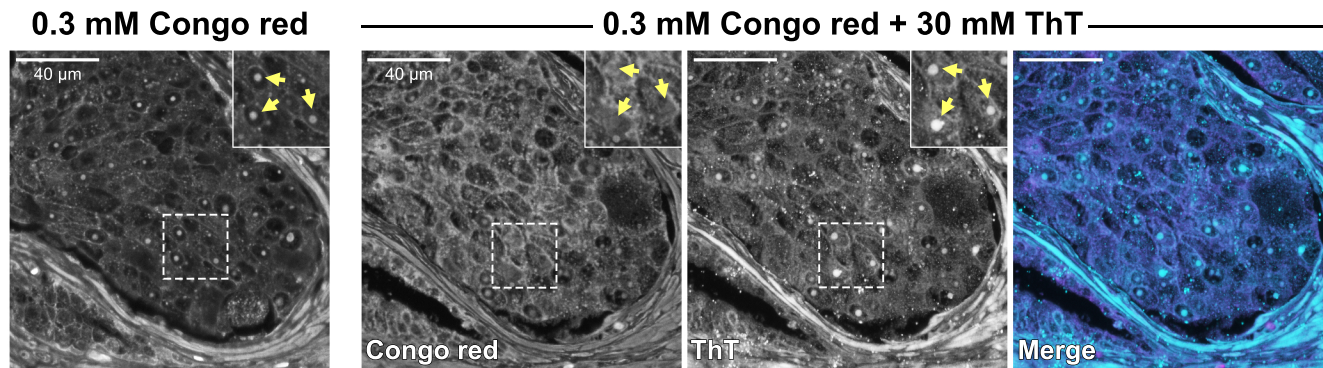

Supplement: S2 Fig — A). FFPE serial sections of a breast invasive ductal carcinoma tumor were incubated with 1X PBS or 20 µg/mL proteinase K for 20 min at 37 ºC and stained for Amyloid-bodies (Amylo-Glo) and nucleoli (B23). B). Polarized light microscopy of a breast invasive ductal carcinoma tumor stained with Congo red. C). FFPE serial sections of a prostate adenocarcinoma tumor stained with 0.3 mM Congo red alone or sequentially stained with 0.3 mM Congo red followed by 30 mM Thioflavin T (ThT). (PDF) [file pone.0353464.s002.pdf]

**A**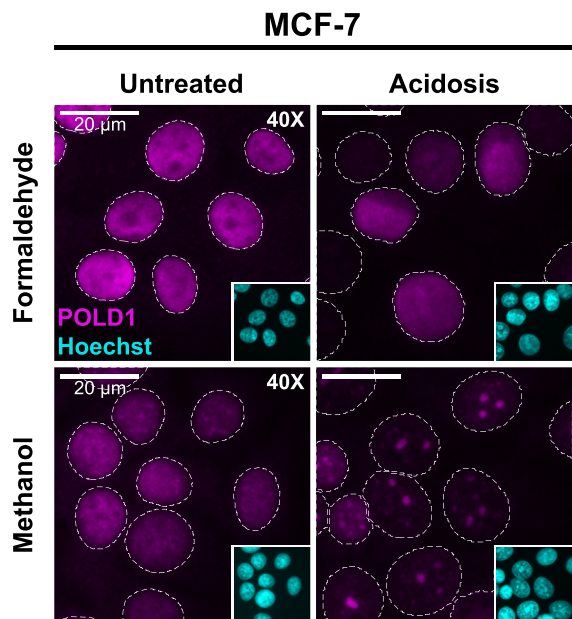**B**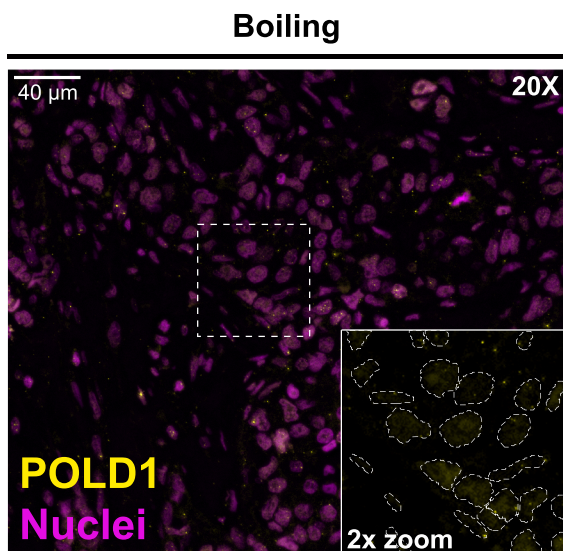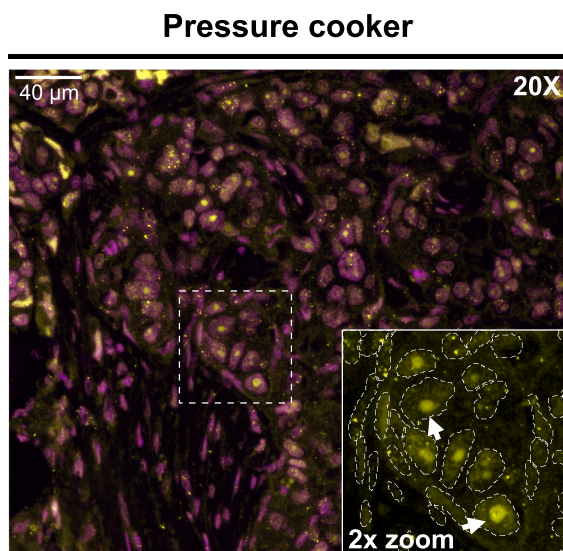

Supplement: S3 Fig — A). Comparison of fixation methods on Amyloid-body protein detection in cultured cell lines. MCF-7 cells were grown in basal growth conditions (21% O2, pH 7.4) or in hypoxia-acidosis (1% O2, pH 6.0) for 3 hours, fixed, and stained for POLD1. B). Comparison between boiling and pressure cooking as antigen retrieval methods to detect the Amyloid-body target protein POLD1 (yellow) in FFPE tissues. (PDF) [file pone.0353464.s003.pdf]

**A**

Amylo-Glo segmentation (ilastik) + postprocessing (cellprofiler) workflow

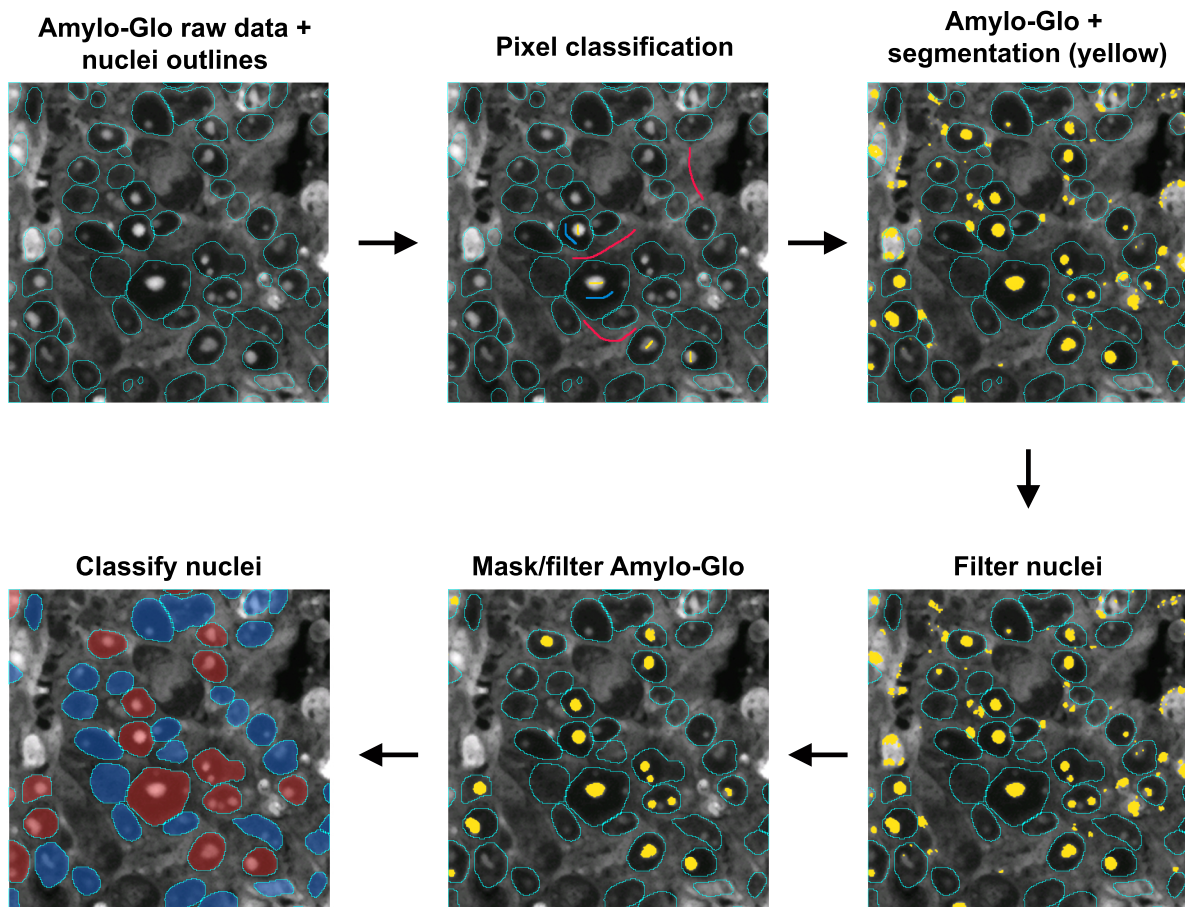**B**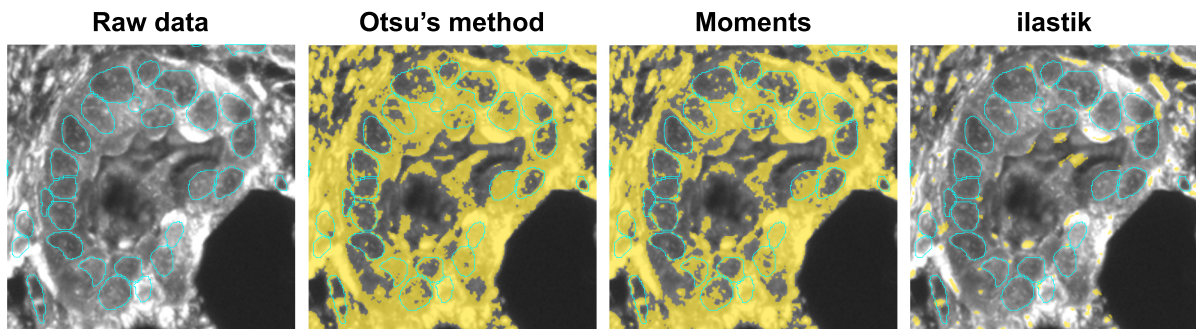

Supplement: S4 Fig — A). Detailed schematic showing how segmentation of Amylo-Glo images was performed in Ilastik. Using the pixel classification toolkit, three classes of pixels were manually defined as the Amyloid-body (yellow line), nucleoplasmic background (blue line), or cytoplasmic background (pink line). After training ~6 images with this classification scheme, the resulting pixel classification model was applied to all remaining images in the data set to produce a combined thresholded image of all three pixel classes. This image was then imported into fiji to isolate the “Amyloid-body” thresholded image to import into CellProfiler for additional processing and quantification. B). Comparison of Amylo-Glo segmentation performed in ilastik versus traditional thresholding algorithms (Otsu’s and moments). Nuclei (cyan outlines) and the thresholding output (yellow) are shown as overlays on the original Amylo-Glo image. The Amylo-Glo example used is a representative image with Amyloid-body negative cells. Compared to the thresholded images produced by Otsu’s method and moments, the ilastik segmentation is more specific and less likely to threshold cytoplasmic signal which may later be detected in the nucleus as false-positive signal. (PDF) [file pone.0353464.s004.pdf]
