# Supplementary material for: Amyloid-bodies in the evolution of malignancies
Source: PLoS One. 2026 Jul 9;21(7):e0353464. doi: 10.1371/journal.pone.0353464 (PMC13349100; doi:10.1371/journal.pone.0353464)
Supplement: S5 Fig — Representative images of tumors which are Amyloid-body negative, strongly positive, or intermediate in phenotype (containing few cells with prominent Amyloid-bodies or a larger number of cells with small and/or low intensity Amyloid-bodies). (PDF) [file pone.0353464.s005.pdf]

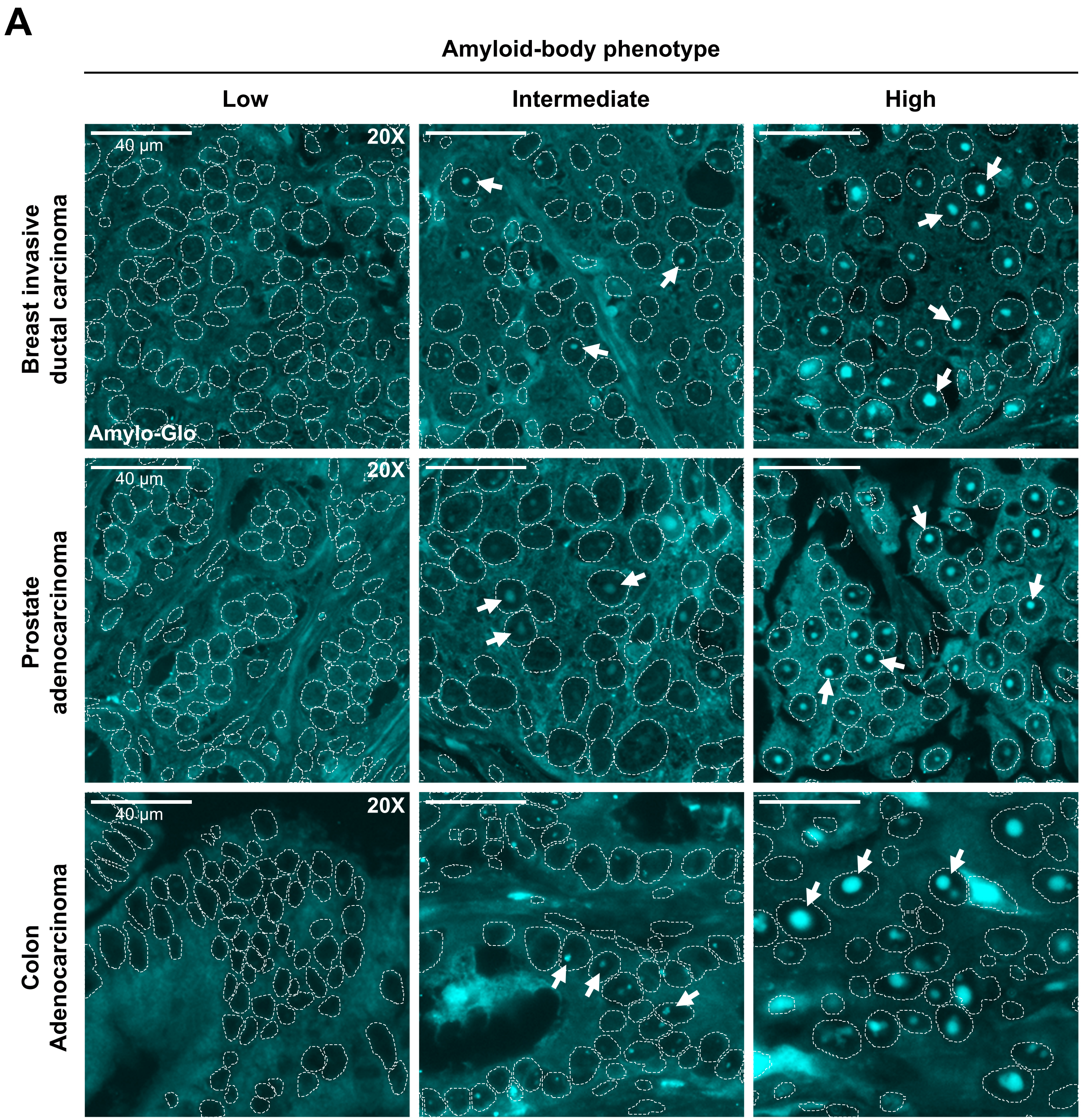
A
Amyloid-body phenotype
Low
Intermediate
High
Breast invasive ductal carcinoma
Prostate adenocarcinoma
Colon Adenocarcinoma
40 µm
20X
Amylo-Glo
